# Approaches to prioritising research for clinical trial networks: a scoping review

**DOI:** 10.1186/s13063-022-06928-z

**Published:** 2022-12-12

**Authors:** Rachael L. Morton, Haitham Tuffaha, Vendula Blaya-Novakova, Jenean Spencer, Carmel M. Hawley, Phil Peyton, Alisa Higgins, Julie Marsh, William J. Taylor, Sue Huckson, Amy Sillett, Kieran Schneemann, Anitha Balagurunanthan, Miranda Cumpston, Paul A. Scuffham, Paul Glasziou, Robert J. Simes

**Affiliations:** 1 National Health and Medical Research Council Clinical Trials Centre (NHMRC CTC), University of Sydney, Sydney, Australia; 2 Centre for the Business and Economics of Health, University of Queensland, Brisbane, Australia; 3 Australian Clinical Trials Alliance (ACTA), Melbourne, Victoria Australia; 4 Australasian Kidney Trials Network (AKTN), Faculty of Medicine, University of Queensland, Brisbane, Australia; 5 Australian and New Zealand College of Anaesthetists (ANZCA), Melbourne, Australia; 6 Australian and New Zealand Intensive Care Research Centre (ANZIC-RC), Monash University, Melbourne, Victoria Australia; 7 Telethon Kids Institute, West Perth, Australia; 8 University of Otago, Rehabilitation Teaching and Research Unit, Dunedin, New Zealand; 9 Australian and New Zealand Intensive Care Society (ANZICS), Camberwell, Victoria Australia; 10 AstraZeneca Australia, Macquarie Park, New South Wales Australia; 11 School of Medicine and Public Health, The University of Newcastle, Newcastle, Australia; 12 Faculty of Health Sciences & Medicine, Bond University, Gold Coast, Australia

**Keywords:** Cost-effectiveness, Clinical trial networks, Prioritisation, Review

## Abstract

**Background:**

Prioritisation of clinical trials ensures that the research conducted meets the needs of stakeholders, makes the best use of resources and avoids duplication. The aim of this review was to identify and critically appraise approaches to research prioritisation applicable to clinical trials, to inform best practice guidelines for clinical trial networks and funders.

**Methods:**

A scoping review of English-language published literature and research organisation websites (January 2000 to January 2020) was undertaken to identify primary studies, approaches and criteria for research prioritisation. Data were extracted and tabulated, and a narrative synthesis was employed.

**Results:**

Seventy-eight primary studies and 18 websites were included. The majority of research prioritisation occurred in oncology and neurology disciplines. The main reasons for prioritisation were to address a knowledge gap (51 of 78 studies [65%]) and to define patient-important topics (28 studies, [35%]). In addition, research organisations prioritised in order to support their institution’s mission, invest strategically, and identify best return on investment. Fifty-seven of 78 (73%) studies used interpretative prioritisation approaches (including Delphi surveys, James Lind Alliance and consensus workshops); six studies used quantitative approaches (8%) such as prospective payback or value of information (VOI) analyses; and 14 studies used blended approaches (18%) such as nominal group technique and Child Health Nutritional Research Initiative. Main criteria for prioritisation included relevance, appropriateness, significance, feasibility and cost-effectiveness.

**Conclusion:**

Current research prioritisation approaches for groups conducting and funding clinical trials are largely interpretative. There is an opportunity to improve the transparency of prioritisation through the inclusion of quantitative approaches.

## Introduction

Clinical trials networks (CTNs) conduct investigator-initiated research and public good trials, largely funded by charities, universities and governments. Examples of CTNs in Australia include the Australian Kidney Trials Network (AKTN https://aktn.org.au/), the Australia and New Zealand College of Anaesthetists clinical trials network (ANZCA https://www.anzca.edu.au/research/anzca-clinical-trials-network) and the Cooperative Trials Group for Neuro-Oncology (COGNO https://www.cogno.org.au/default.aspx). Example CTNs in Europe include the European Society of Anaesthesiology and Intensive Care (ESAIC https://www.esaic.org/research/clinical-trial-network/) and in the USA include the HIV Prevention Trials Network (HPTN https://www.hptn.org/). However, with limited available resources including trained research personnel, trial participants and funds, decisions need to be made about which trials are a priority. The desired result of successful prioritisation is funded trials that generate important information, help inform clinical and policy decision-making and improve health outcomes. In reality, research prioritisation is not easy, and many organisations wrestle with competing criteria and the multiple interests of stakeholder groups.

Three main approaches to prioritisation have emerged in health and medicine research, namely interpretive, quantitative and blended methods. Interpretive approaches utilise consensus views of informed participants and include James Lind Alliance (JLA) and Delphi surveys [1, 2]. These approaches can reflect emerging patterns in the future and engage consumers; however, they do not provide methodology for identifying participants, often lack criteria transparency and have the potential for investigators and facilitators to bias opinions. Quantitative approaches utilise epidemiological, clinical or economic data. Examples include burden of disease, prospective payback and value of information (VOI) analyses [3, 4]. These approaches provide an objective assessment of value for money; however, they do not consider other criteria such as equity and broad stakeholder’s involvement; furthermore, they can be technically demanding. Blended approaches utilise and combine both interpretive and quantitative assessments and include the Child Health Nutrition Research Initiative (CHNRI) and multi-criteria decision analysis (MCDA [5, 6].

The aim of this scoping review was to identify approaches for priority setting in health and medical research useful to clinical trial networks (CTNs) in Australia and internationally and research funders. Specifically to answer the following research questions: what models, approaches or methods are used by CTNs to prioritise clinical trials; how have these models, approaches or methods been developed and validated; and what is the best practice for prioritising clinical trials? The findings will then be used to develop best practice guidance for CTNs and research funders.

## Methods

A scoping review of published literature and working documents, as well as websites from research funding organisations and CTNs, was undertaken to identify research prioritisation tools and criteria. Digital databases including Ovid MEDLINE, Embase and the WHO library database (WHOLIS) were searched for publications about guidelines for prioritising research questions relevant to CTNs. Search terms included ([prioritization OR prioritisation OR setting priorities OR priority setting OR research priority*] AND [clinical trials OR clinical trial networks OR clinical trial group]). The search was limited to studies in English published from year 2000 onwards. The search was updated on 30 January 2020.

Titles and abstracts were screened, and eligible studies were selected by a single reviewer (VBN) for the following inclusion criteria: original studies, systematic reviews, guidelines, recommendations, and tools for research prioritisation. Both qualitative and quantitative methods of prioritisation were accepted. Studies not relevant to CTNs, duplicate publications, guidelines written from the perspective of funders, opinion articles, letters to editors and abstracts only were excluded. A manual search of key references cited in the retrieved papers and reports was also undertaken to identify additional publications not encountered by the electronic searches. A second reviewer (RLM) was consulted when in doubt regarding study selection, and any discrepancies were resolved by consensus with a third reviewer (MC).

A second search of key Australian and international CTNs/clinical disciplines/clinical specialties websites was then undertaken. Organisations were selected by the author team as likely to provide guidance on prioritisation and selection of clinical trials, and websites were searched by two authors (VBN, AB). Searched websites are listed in the Appendix. Searching included exploration of the website menu structure for relevant documents and searching within the sites using the terms “clinical trials”, “priorities”, “prioritisation” or “prioritization” (depending on the nationality of the website).

The following types of documents were selected for inclusion: guidance on prioritisation; case studies or examples of prioritisation exercises that reported the methods used; guidance on criteria for the assessment, selection or prioritization of clinical trials (e.g. for funding purposes). Documents that did not constitute current guidance or were superseded by later versions of current guidance (e.g. prioritisation processes to inform past priorities or strategic plans or discussion documents that appeared to be older than current guidance), were excluded. Documents with URLs that were no longer accessible in January 2020 were also excluded.

Data from studies and websites were extracted and tabulated into an Excel file according to a predefined codebook. Data extraction variables comprised author name, author group (e.g. CTN, funder), clinical discipline, country, year of publication, participants or stakeholders in the prioritisation process (e.g. health professionals, researchers, policy/decision makers, funders, patients, carers/consumers), intended audience (e.g. government/policymakers, clinicians, researchers, funders, the public), brief reason for prioritisation (e.g. knowledge gap, important to patients, return on investment, feasibility of methodology), type of research (e.g. trials), research prioritisation tools (e.g. Delphi, CHNRI, JLA, payback, MCDA, forced ranking, workshop/consensus meeting, other), prioritisation method (e.g. quantitative scoring, nominal group technique, weighted scores, monetary, other), research prioritisation criteria (e.g. relevance, appropriateness, significance, feasibility, cost-effectiveness [7]) and the URLs (for websites). Data from published articles and websites were summarised and tabulated separately. Critical appraisals of included studies, guidance documents or websites were not undertaken. Reporting of this scoping review was consistent with items in the PRISMA-ScR checklist [8].

## Results

### Literature search

**Fig. 1:**
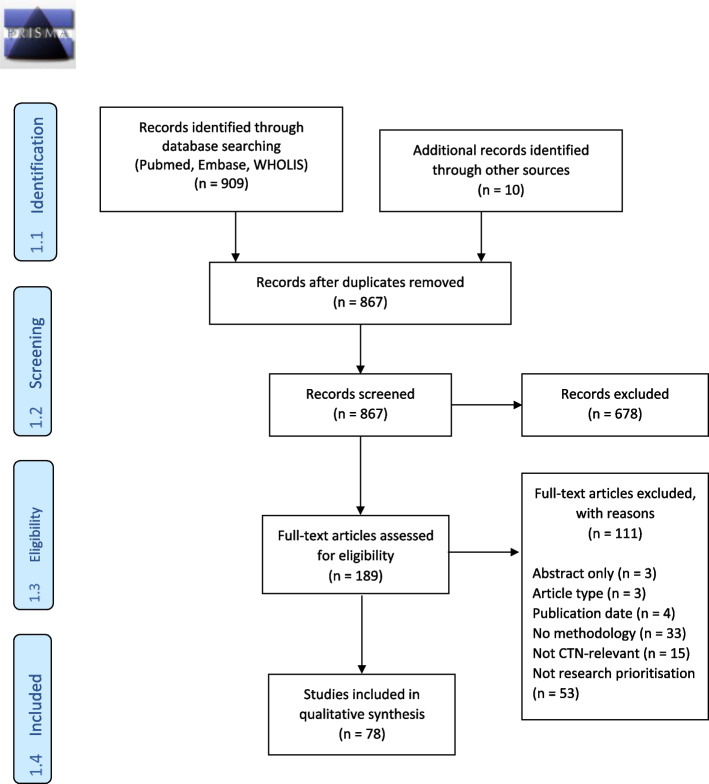
PRISMA 2009 diagram

The results of the literature search and study selection process are depicted in Fig. 1. A table of the seventy-eight primary studies included in this review is presented in Table 1.

**Table 1:** Included studies

Citation	Author group (CTN, funder)	Clinical discipline	Participants/Stakeholders
*Africa*			
Folayan, Haire [9]	Institute of Public Health, Obafemi Awolowo University, Nigeria	Infectious diseases (outbreaks)	Bioethicists, social scientists, ethics committee members, community members
*Australia and New Zealand*			
Middleton, Piccenna [10]	National Trauma Research Institute and Australian and New Zealand Spinal Cord Injury Network (ANZSCIN) Funded by Victorian Transport Accident Commission and ANZSCIN	Spinal cord injury	Clinicians, researchers, advocacy organisations, health system managers, policy makers, funding agencies
Sangvatanakul, Hillege [11]	Research institutes, universities, Nursing and Midwifery Australia, National Centre for Clinical Outcomes Research (NaCCOR), National Stroke Foundation…	Stroke	Stroke survivors, carers
Sawford, Dhand [12]	University of Western Sydney, contracted by Rural Industries Research and Development Corporation Funded by National Hendra Virus Research Program (Commonwealth of Australia, State of New South Wales, State of Queensland)	Infectious diseases/ Zoonosis	Policy developers and implementers in key government agencies in all states and territories Known experts engaged in a range of Hendra virus-related activities Research leaders in charge of National Hendra Virus Research Program funded projects Members of the Intergovernmental Hendra Virus Taskforce Public health leaders in Hendra virus-affected states
Thom, Keijzers [13]	Australian College for Emergency Medicine (ACEM) Clinical Trials Network	Emergency medicine	ACEM fellows, trainees, senior national and international researchers
Tong, Crowe [14]	Funded by the National Health and Medical Research Council (NHMRC), University of Sydney, Kidney Health Australia	Nephrology (chronic kidney disease (CKD))	Patients with CKD (CKD stages 1- 5, 5D, or 5T), family caregivers, or health professionals with experience in CKD (nephrologists, surgeons, nurses, allied health professionals, and researchers)
Taylor and Green [15]	Australia & New Zealand Musculoskeletal Clinical Trials Network (ANZMUSC)	Rehabilitation	Health professionals from various disciplines, consumers of healthcare services, funders of research and healthcare services
*North America – Canada*			
Barnieh, Jun [16]	University of Calgary	Nephrology (dialysis)	Patients, caregivers, clinicians
Hayes, Bassett-Spiers [17]	The Ontario Neurotrauma Foundation (ONF) International Expert Panel	Spinal cord injury (SCI)/Urology	Experts in physiatry, urology, nursing, microbiology, physiology; person with SCI; executive representatives of ONF
Lavigne, Birken [18]	TARGet Kids! (The Applied Research Group for Kids) primary care research network	Paediatric preventive care	Parents, clinicians
Manns, Hemmelgarn [19]	Kidney Foundation of Canada Funded by Canadian Institutes of Health Research (CIHR) (grant)	Nephrology (dialysis)	Patients, carers, clinicians
Ota, Cron [20]	Childhood Arthritis and Rheumatology Research Alliance (CARRA) - investigator-initiated research network	Paediatric rheumatology	Paediatric rheumatology experts across Canada and the USA
Restall, Carnochan [21]	Canadian Institutes of Health Research (CIHR)	HIV/AIDS	People living with HIV, researchers, service providers, leaders in AIDS service or related organisations and policy makers
Schneider, Evaniew [22]	McMaster University Funded by the McMaster Surgical Associates Innovation Grant	Orthopaedic oncology	Clinician-scientists (interested or participating in a trial, professional societies members), representatives from patient advocacy groups. Representation of geographical, stakeholder and career stage groups.
Sivananthan and Chambers [23]	Ontario Research Coalition of Institutes/Centres on Health and Aging (ORC)	Health and aging	Researchers, policy makers, caregivers
Wu, Bezjak [24]	National Cancer Institute of Canada (NCIC) Clinical Trials Group	Oncology	Researchers, radiation oncologists Opinion leaders, researchers, methodologists
*North America – USA*			
Al-Khatib, Gierisch [25]	Duke University Evidence Synthesis Group Funded by the Patient- Centered Outcomes Research Institute (PCORI)	Cardiovascular	Clinical experts, researchers, funding agencies, healthcare decision-makers, policymakers, consumer and patient advocacy groups
Ardoin, Daly [26]	Lupus Foundation of America (LFA) Childhood Arthritis and Rheumatology Research Alliance (CARRA)	Paediatric rheumatology	Paediatric clinicians and investigators in rheumatology, nephrology and dermatology
Bennette, Veenstra [27]	Southwest Oncology Group (SWOG) – Clinical Trial Cooperative Group Funded by Patient-Centered Outcomes Research Institute (PCORI)	Oncology	Members of SWOG, including clinical trialists, clinicians, statisticians, and patient advocates and/or members who have a vested interest in the outcomes of this work
Bousvaros, Sylvester [28]	Challenges in Pediatric Inflammatory Bowel Disease (IBD) Study Groups	Paediatric Inflammatory Bowel Disease	Investigators with expertise in paediatric IBD: paediatricians, internists, basic scientists, clinical investigators, and members of the administrative staff and board of the Crohn's and Colitis Foundation of America
Carlson, Kim [29]	Southwest Oncology Group (SWOG) Funded by Patient-Centered Outcomes Research Institute (PCORI)	Oncology	SWOG
Duong, Schempp [30]	United States Army/ TriService Nursing Research Program (TSNRP)	Military nursing	TSNRP director, TSNRP Advisory Council, military nursing researchers, clinical leaders
Esmail, Roth [31]	Center for Comparative Effectiveness Research in Cancer Genomics (CANCERGEN)	Cancer genomics	CANCERGEN External Stakeholder Advisory Group (ESAG): professional patient/consumer advocates, payers, clinicians, policymakers/regulators, the life sciences and diagnostic industry
Fochtman and Hinds [32]	Association of Pediatric Oncology Nurses	Paediatric oncology	Nurse experts
Henkle, Aksamit [33]	Oregon Health & Science University Supported by Patient-Centered Outcomes Research Institute (PCORI)	Infectious diseases	Nontuberculous Mycobacteria (NTM) Research Consortium: clinical experts, researchers, patients, caregivers, patient advocates
Henkle, Aksamit [34]	Funded by Patient-Centered Outcomes Research Institute (PCORI)	Pneumology	Clinical research experts, patient advisory panel, representatives from two key patient advocacy organisations
Higginbotham [35]	Society of Family Planning	Family planning	Family planning researchers and academics
Roach, Abreu [36]	2015 Sturge-Weber Syndrome Research Workshop Funded by the National Institutes of Health (NIH)	Sturge-Weber Syndrome (Neurology, Ophthalmology, Dermatology)	Clinical and translational researchers
Safdar and Greenberg [37]	Yale School of Medicine and USF Morsani College of Medicine Funded by National Institute of Neurological Disorders and Stroke (NINDS) and National Institutes of Health (NIH) Supported by Patient-Centered Outcomes Research Institute (PCORI)	Emergency Medicine	Researchers, clinicians, health care providers, patients, representatives of federal agencies, policymakers
Saldanha, Dickersin [38]	Johns Hopkins Funded by the National Institutes of Health (NIH) and Cochrane Eyes and Vision	Ophthalmology	International (21 countries) clinicians managing patients with Dry Eye
Thariani, Wong [39]	Center for Comparative Effectiveness Research in Cancer Genomics (CANCERGEN)	Oncology (cancer genomics)	Representatives from patient- advocacy groups, payers, test developers, regulators, policymakers, and community-based oncologists
Vickrey, Brott [40]	National Institutes of Health (NIH)/ National Institute of Neurological Disorders and Stroke (NINDS)	Stroke	Scientific experts, stroke advocates, stroke association representatives
*Europe*			
Aliberti, Masefield [41]	European Multicentre Bronchiectasis Audit and Research Collaboration (EMBARC) European Respiratory Society (ERS) Clinical Research Collaboration Endorsed by ERS	Pneumology	EMBARC Roadmap Study Group: clinicians, patients, and carers
Forsman, Wahlbeck [42]	ROAdmap for Mental health Research in Europe (ROAMER) Consortium	Mental health	Experts
van der Feltz- Cornelis, van Os [43]	ROAdmap for MEnatal health Research and well-being in Europe (ROAMER) Funded by the European Commission's 7th Framework Programme	Mental health	Experts in the field of clinical mental health research: psychiatrists, psychologists, general physicians, occupational physicians
*Europe – The Netherlands*			
de Graaf, Postmus [44]	Department of Epidemiology, University of Groeningen	Diabetes	Not applicable (theoretical exercise) Expert opinion for ordinal ranking of decision alternatives
*Europe – United Kingdom*			
Aldiss, Fern [45]	The Teenage and Young Adult Cancer Priority Setting Partnership (PSP) Funded by the Teenage Cancer Trust, CLIC Sargent, Children with Cancer UK	Oncology	Young people with current or previous cancer diagnosis, their families, friends, partners, and professionals who work with this population
Andronis, Billingham [46]	National Institute for Health Research (NIHR)	Oncology	2 case studies (research grant proposals for clinical trials)
Boney, Bell [47]	National Institute for Academic Anaesthesia (NIAA) Health Services Research Centre	Anaesthesia and perioperative care	Professionals, patients/carers
Cox, Arber [48]	UK Oncology Nursing Society	Oncology nursing	Nurses, patients
Deane, Flaherty [49]	University of East Anglia and University of Birmingham Funded by Parkinson's UK	Neurology (Parkinson's)	People with Parkinson’s (PwP); carers and former carers; family members and friends; healthcare and social care professionals who work, or have worked, with people living with the condition. Non- clinical researchers and employees of pharmaceutical or medical devices companies were excluded from the survey.
Fleurence [50]	York Trials Unit	Methodology (clinical trials)/ osteoporosis and wound care	Not applicable (theoretical exercise)
Gadsby, Snow [51]	University of Warwick Partnership: Juvenile Diabetes Research Foundation, Insulin Dependent Diabetes Trust, Diabetes Research Network, Diabetes UK, Scottish Diabetes Research Network, UK Database of Uncertainties in the Effects of Treatments, the James Lind Alliance, and NHS Evidence—diabetes Funded by Insulin Dependent Diabetes Trust	Diabetes	Patients, carers, health professionals
Hall, Mohamad [52]	National Institute for Health Research (NIHR), National Institute for Clinical Excellence (NICE), scientific and patient societies Funders: British Tinnitus Association, NIHR, Judi Meadows Memorial Fund	Neurology - tinnitus	Clinicians, persons with tinnitus, researchers, James Lind Alliance representative, NICE representative Professional bodies, charities, advocators for people with tinnitus, support groups, hospital centres, commercial organizations
Hart, Lomer [53]	British Society of Gastroenterology, Funded by Crohn's and Colitis UK	Gastroenterology	Healthcare professionals (nurses, gastroenterologists, dietitians), patients, carers
Heazell, Whitworth [54]	Tommy's, Maternal and Fetal Health Research Centre University of Manchester	Obstetrics	Representatives of professional and parents' organisations (direct/indirect experience with stillbirth)
Howell, Pandit [55]	National Institute for Academic Anaesthesia (NIAA) Research Council	Anaesthesia and perioperative medicine	Fellows of the Royal College of Anaesthetists (RCA), members of the Association of Anaesthetists of Great Britain and Ireland (AAGBI), lay representatives (Patient Liaison Group of the RCA)
Ingram, Abbott [56]	Funded by the UK Dermatology Clinical Trials Network	Dermatology	Patients, carers, clinicians
Kelly, Lafortune [57]	Alzheimer's Society	Neurology / dementia	People with dementia, carers, relatives, health and care professionals
Knight, Metcalfe [58]	Funded by the National Institute for Health Research (NIHR)	Nephrology (transplant)	Patients, carers, donors, clinicians, nurses, scientists
Macbeth, Tomlinson [59]	British Hair and Nail Society Funded by Alopecia UK	Dermatology	People with hair loss, carers, relatives, healthcare professionals, scientific societies' representatives
McKenna, Griffin [60]	University of York Supported by Patient-Centered Outcomes Research Institute (PCORI)	Traumatology (brain injury)	Not applicable (case study)
Morris, Simkiss [61]	British Academy of Childhood Disability	Childhood neurodisability	Young people with disabilities, parent carers, clinicians, charity representatives
Owens, Ley [62]	Devon Partnership National Health Service (NHS) Trust	Mental health	Mental health service users Informal carers Mental health practitioners Service managers
Perry, Wright [63]	British Society for Children's Orthopaedic Surgery (BSCOS)	Paediatric Orthopaedics	Surgeons - members of BSCOS
Pollock, St George [64]	Nursing Midwifery and Allied Health Professions (NMAHP) Research Unit Funded by the Scottish Government	Stroke	Stroke survivors, caregivers, health professionals
Rangan, Upadhaya [65]	National Institute for Health Research (NIHR) Funded by the British Elbow and Shoulder Society, British Orthopaedic Association	Orthopaedics	Patients, carers, medical doctors, nurses, allied health professionals, general practitioners
Rowat, Pollock [66]	Scottish Stroke Nurses Forum (SSNF)	Stroke (nursing)	Stroke nurses (registered, unregistered, students) members of the SSNF
Rowe, Wormald [67]	Fight for Sight, College of Optometrists, Royal College of Ophthalmologists Funded by the National Institute for Health Research (NIHR)	Ophthalmology	Patients, relatives, carers, eye health professionals
Shepherd, Wood [68]	South East Wales Trials Unit, Centre for Trials Research, Cardiff University	Aged care	Care home staff (nursing and residential care)
Stephens, Whiting [69]	Institute of Clinical Trials & Methodology	Oncology (mesothelioma)	Patients, carers, health professionals, support organisations
van Middendorp, Allison [70]	Funded by the National Institute for Health Research (NIHR) – Oxford Biomedical Research Group	Spinal cord injury	Consumer organisations, healthcare professional societies and caregivers
Wan, Beverley-Stevenson [71]	University of Manchester Funded by NIHR	Oncology	Patients, carers, healthcare professionals
Willett, Gray [72]	Funded by AO UK Research Group	Orthopaedic trauma	AO UK faculty members: orthopaedic surgeons and operating room nurses
*India*			
Arora, Mohapatra [73]	Inclen Trust Intl Indian Council of Medical Research (ICMR)	Maternal, newborn, child health and nutrition	Researchers, professionals, public health functionaries, policy makers, communities and their leadership, civil society, donor agencies and industries Exclusively Indian nationals
Ravindran and Seshadri [74]	Institute for Medical Sciences & Technology, Trivandrum Part of Closing the Gap project Supported by International Development Research Centre, Canada	Health equity	Researchers (public health - health systems researchers, epidemiologists, social science, anthropology), practitioners: policymakers, programme managers, advocates, activists
*International*			
Allotey, Matei [75]	Queen Mary University of London Department of Reproductive Health and Research, World Health Organization (WHO)	Maternal and perinatal health	Healthcare providers, academics, lay representatives, public health specialists, policy makers. Clinicians (80%, 127/159), made up of obstetricians (68%, 86/127); neonatologists (24%, 30/127); nurses/midwives (7%, 9/127) and general practitioners (2%, 2/127). Researchers, epidemiologists, consumers, policy makers and representatives of non- governmental organizations (NGOs) and funding bodies
Bahl, Martines [76]	Department of Child and Adolescent Health & Development, World Health Organization (WHO)	Newborn health	Investors, policymakers, technical experts, other stakeholders
Brown, Hess [77]	University of California Davis Funded by the Child Health and Nutrition Research Initiative (CHNRI)	Paediatric nutrition	Leading experts in zinc research
Brundin, Barkerb [78]	Linked Clinical Trials International Committee	Neurology - Parkinson's	International committee of experts Representatives of key funding bodies (as observers)
Foster, Dziedzic [79]	Arthritis Research Campaign National Primary Care Centre, Keele University Clinical Trials Thinktank	Musculoskeletal disorders	Researchers, patient representatives Round 2 - researchers, practitioners, educators, managers
Prescott, Iwashyna [80]	International Sepsis Forum	Infectious diseases	Healthcare professionals, researchers, patient representatives
Robinson, Lorenc [81]	British Acupuncture Council	Traditional Chinese Medicine (TCM)	TCM acupuncturists
Rowbotham, Smith [82]	University of Nottingham	Pneumology (cystic fibrosis)	Patients and clinical community
Ruhl, Sadreameli [83]	The American Thoracic Society	Pneumology (sickle cell lung disease)	Multidisciplinary - paediatric and adult haematologists, pneumologists, emergency medicine physicians, patient advocate, librarian
Viergever, Olifson [84]	Bruyere Evidence-Based Guidelines Symposium	Clinical pharmacology (deprescribing)	Researchers, educators, clinicians, patient advocates, guideline developers, policy makers, other stakeholders
Viergever, Olifson [85]	World Health Organization (WHO)	Health research	Expert staff in WHO and selection of international research organisations experienced in health research priority setting
Yu, Li [86]	Johns Hopkins Bloomberg School of Public Health	Ophthalmology	Clinicians

**Fig. 2:**
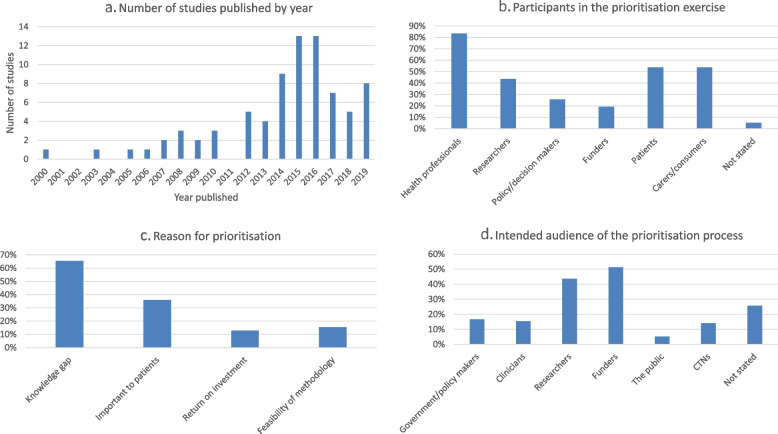
Prioritisation study numbers, participants, reason and intended. **a** Number of studies published by year. **b** Participants in the prioritisation exercise. **c** Reason for prioritisation. **d** Intended audience of the prioritisation process

Most research prioritisation exercises were conducted either in Europe (*n* = 32; 41%) or North America (*n* = 25; 32%); six prioritisation studies (8%) originated in Australia and New Zealand. Two studies were conducted in South Asia (India; 3%), one in South-East Asia (1%), one in Africa (1%) and 11 studies were international (14%). Included studies were published between 2000 and 2019 (see Fig. 2a). Clinical specialties most frequently involved in research prioritisation were oncology (*n* = 11 studies; 14%), neurology (*n* = 11; 14%), paediatrics (*n* = 8; 10%), maternal and child health (*n* = 4; 5%), infectious diseases and HIV/AIDS (*n* = 5; 6%), nephrology (*n* = 4; 5%), respiratory medicine (*n* = 4; 5%), mental health (*n* = 3; 4%) and ophthalmology (*n* = 3; 4%).

The stakeholders most frequently involved in the prioritisation process were health professionals (*n* = 65 studies; 83.3%), patients and carers/consumers (each *n* = 42 studies; 53.8%), researchers (*n* = 34 studies, 44%), policy or decision makers (*n* = 20 studies, 26%), and funders (*n* = 15 studies, 19%; see Fig. 2b). Stakeholders were not stated in four studies (5%).

The reasons for conducting the research prioritisation exercise included a knowledge gap in 51 studies (65%), ascertaining what was important to patients in 28 studies (36%), assessing the feasibility of a particular prioritisation methodology in 12 studies (15%) and estimating a return on investment in 10 studies (13%; see Fig. 2c; Table 2).

**Table 2:** Prioritisation methodologies

Citation	Type of research	Reason for prioritisation				Tool, model or approach used for prioritisation	Prioritisation method
		Knowledge gap	Important to patients	Return on investment	Feasibility of methodology		
***Quantitative Approach***							
Andronis 2016 [46]	Clinical trials				Y	Payback/VOI	Monetary
Bennette 2016 [27]	Clinical trials				Y	VOI	Monetary
Carlson 2018 [29]	Clinical trials				Y	VOI	Monetary
Fleurence 2007 [50]	Trials				Y	Payback/VOI	Monetary
McKenna 2016 [60]	Clinical trials				Y	VOI	Monetary
Robinson 2012 [81]	Clinical trials	Y				Other	Quantitative scoring
***Interpretative Approach***							
Aldiss 2019 [45]	Not stated		Y			JLA	Not stated
Aliberti 2016 [41]	Clinical trials Translational research Collaborative working	Y				Delphi	Quantitative scoring
Al-Khatib 2015 [25]	Systematic reviews, trials, observational studies (horizon scanning)	Y				Forced ranking	Forced ranking
Barnieh 2015 [16]	Not stated		Y			JLA	Nominal group technique
Boney 2015 [47]	Not stated	Y	Y			JLA	Weighted scores
Bousvaros 2006 [28]	Not stated	Y		Y		Workshop/consensus meeting	Not stated
Brundin 2013 [78]	Clinical trials					Other	Not stated
Comi 2016 [87]	Clinical trials			Y		Workshop/consensus meeting	Not stated
Cox 2017 [48]	Not stated	Y	Y			Delphi	Quantitative scoring
Deane 2014 [49]	Not stated	Y	Y			JLA	Quantitative scoring
Duong 2005 [30]	Not stated	Y				Workshop/consensus meeting	Not stated
Esmail 2013 [31]	Comparative effectiveness research				Y	Delphi	AHRQ criteria
Fochtman 2000 [32]	Not stated	Y				Delphi	Quantitative scoring
Folayan 2018 [9]	Clinical trials	Y				Delphi	Not stated
Forsman 2015 [42]	Not stated	Y				Delphi	Quantitative scoring
Foster 2009 [79]	Clinical trials	Y				Delphi	Quantitative scoring/Nominal group technique
Gadsby 2012 [51]	Not stated	Y				JLA	Quantitative scoring
Hall 2013 [52]	Not stated	Y	Y			JLA	Weighted scores
Hart 2017 [53]	Not stated	Y				JLA	Quantitative scoring
Hayes 2007 [17]	Late-stage animal or early-stage human clinical trials			Y		Delphi	Quantitative scoring
Heazell 2015 [54]	Not stated	Y	Y			JLA	Quantitative scoring
Henkle 2016 [33]	Not stated		Y			Workshop/consensus meeting	Not stated
Henkle 2018 [34]	Clinical trials	Y	Y			Other	Not stated
Howell 2012 [55]	Not stated			Y		Other	Quantitative scoring
Ingram 2014 [56]	Not stated	Y	Y			JLA	Quantitative scoring/ Nominal group technique
Kelly 2015 [57]	Not stated	Y				JLA	Quantitative scoring/ Nominal group technique
Knight 2016 [58]	Not stated	Y	Y			JLA	Quantitative scoring/ Nominal group technique
Lavigne 2017 [18]	Not stated	Y	Y			Other	Quantitative scoring/ Nominal group technique
Macbeth 2017 [59]	Not stated	Y	Y			JLA	Quantitative scoring/Nominal group technique
Manns 2014 [19]	Not stated	Y	Y			JLA	NGT
Middleton 2015 [10]	Trials	Y		Y		Other	Not stated
Morris 2015 [61]	Not stated		Y			JLA	Quantitative scoring/Nominal group technique
Ota 2008 [20]	Clinical trials			Y		Delphi	Quantitative scoring
Owens 2008 [62]	Not stated	Y	Y			Delphi	Quantitative scoring
Perry 2018 [63]	Clinical trials (clinical effectiveness)	Y				Delphi	Quantitative scoring
Pollock 2014 [64]	Not stated		Y			JLA	Quantitative scoring
Prescott 2019 [80]	Clinical trials, cohorts	Y	Y			Workshop/consensus meeting	Quantitative scoring
Rangan 2016 [65]	Not stated	Y				JLA	Red-amber-green light
Ravindran 2018 [74]	Not stated	Y				Workshop/consensus meeting	Not stated
Restall 2016 [21]	Not stated		Y			Other	Dotmocracy
Rowat 2016 [66]	Not stated	Y	Y			JLA	Quantitative scoring/ Nominal group technique
Rowbotham 2019 [82]	Clinical trials		Y			JLA	Not stated
Rowe 2014 [67]	Not stated	Y	Y			JLA	Quantitative scoring/ Nominal group technique
Ruhl 2019 [83]	Randomised controlled trials, Longitudinal studies	Y				Workshop/consensus meeting	Not stated
Saldanha 2017 [38]	Clinical research	Y				Delphi	Quantitative scoring
Sawford 2014 [12]	Longitudinal cohort study		Y			Delphi	Quantitative scoring
Shepherd 2017 [68]	Not stated	Y				Delphi	Quantitative scoring
Sivananthan 2013 [23]	Not stated		Y			Delphi	Quantitative scoring
Stephens 2015 [69]	Not stated	Y				JLA	Not stated
Thariani 2012 [39]	Comparative effectiveness research				Y	Delphi	Quantitative scoring
Thompson 2019 [84]	Clinical trials, cohorts	Y				Other	None
van der Feltz-Cornelis 2014 [43]	Clinical research	Y				Other	Quantitative scoring
van Middendorp 2016 [70]	Not stated	Y	Y			JLA	Quantitative scoring
Vickrey 2013 [40]	Not stated	Y				Delphi	Not stated
Wan 2016 [71]	Not stated	Y	Y			JLA	Quantitative scoring
Willett 2010 [72]	RCTs	Y		Y		Delphi	Quantitative scoring
Wu 2003 [24]	Clinical trials			Y		Workshop/consensus meeting	Not stated
***Blended Approach***							
Allotey 2019 [75]	Clinical trials, IPDM	Y				Other	Quantitative scoring
Ardoin 2019 [26]	Clinical trials	Y				Other	Not stated
Arora 2017 [73]	Not stated	Y		Y	Y	CHNRI	Weighted scores
Bahl 2009 [76]	Funding agencies and investigators	Y		Y		CHNRI	Weighted scores
Brown 2008 [77]	Not stated	Y				CHNRI	Quantitative scoring
de Graaf 2015 [44]	Translational biomedical research				Y	MCDA	Quantitative scoring
Higginbotham 2015 [35]	Not stated	Y				CHNRI	Weighted scores
Safdar 2014 [37]	Not stated	Y				Workshop/consensus meeting	Quantitative scoring
Sangvatanakul 2010 [11]	Not stated		Y		Y	Other/Delphi	Quantitative scoring
Schneider 2016 [22]	International clinical trials	Y		Y		Delphi	Quantitative scoring
Taylor 2019 [15]	Review topics	Y				MCDA	Quantitative scoring
Thom 2014 [13]	Clinical research				Y	Workshop/consensus meeting	Weighted scores
Tong 2015 [14]	Not stated	Y	Y			Workshop/consensus meeting	Quantitative scoring
Yu 2015 [86]	Comparative effectiveness study Reviews RCTs	Y				Other	Quantitative scoring
***Other***							
Viergever 2010 [85]	Not stated				Y	Other	Not stated

*AHRQ* Agency for Healthcare Research and Quality, *CHNRI* Child Health and Nutrition Research Initiative, *IPDM* Individual patient data meta-analyses, *JLA* James Lind Alliance, *MCDA* Multicriteria decision analysis, *RCT* Randomised controlled trial

The intended audience for the outcomes of the prioritisation exercise were the funders in 40 studies (51%), researchers in 34 studies (44%), government or policymakers in 13 studies (17%), clinicians in 12 studies (15%), CTNs in 11 studies (14%), the general public in 4 studies (5.1%) and not stated in 20 studies (26%; see Fig. 2d).

**Fig. 3:**
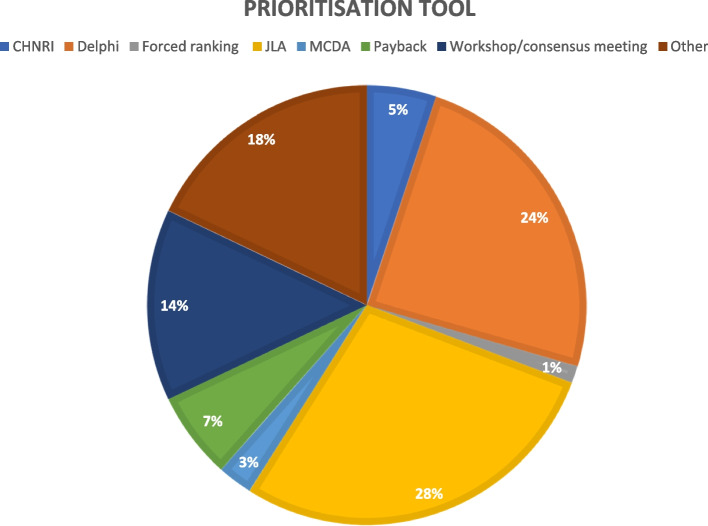
Prioritisation tools used. CHNRI, Child Health and Nutrition Research Initiative; JLA, James Lind Alliance; MCDA, Multiple-criteria Decision Analysis

A table of the prioritisation approaches is presented in Table 2. Fifty-seven studies used interpretative prioritisation approaches (73%), 14 studies used blended approaches (18%) and six studies used quantitative approaches (8%). Twenty-two studies used the JLA prioritisation tool or a modification thereof (28%), 19 studies used the Delphi methodology (24%) and 11 studies used a workshop or consensus meeting to establish their priorities (18%; see Fig. 3; Table 2). The “Payback” category included quantitative methods such as prospective payback of research (PPoR), expected value of information (EVI), return on investment (ROI) and the “Other” category included methods such as online surveys/questionnaires, focus groups, World Café and mixed methods. Forty-five studies (58%) employed quantitative scoring as a prioritisation method, frequently in the form of nominal group technique (*n* = 11 studies; 14%). Six studies used weighted scores (8%) and five studies used monetary value (6%). One study each used Agency for Healthcare Research and Quality (AHRQ) criteria, Dotmocracy, forced ranking, red-amber-green light and no prioritisation (1%).

Over two-thirds of the identified studies (*n*=53, 68%) did not describe any formal prioritisation criteria. In those that did describe prioritisation criteria, multiple criteria were mentioned. Relevance (i.e. why should we do it? including the burden of disease, equity, and knowledge gaps) was cited in 14 of the 78 included studies (18%). Seven studies (9%) cited criteria related to appropriateness (i.e. should we do it? including scientific rigour and suitability to answer the research question); 17 studies (22%) considered criteria related to significance of research outcomes (i.e. what will we get out of it? including impact, innovation, capacity building); 12 studies (15%) cited feasibility among their prioritisation criteria (i.e. can we do it? including team quality and research environment). Cost-effectiveness was considered by fifteen studies (19%). Five studies cited other prioritisation criteria (6%).

### Organisational websites

**Fig. 4:**
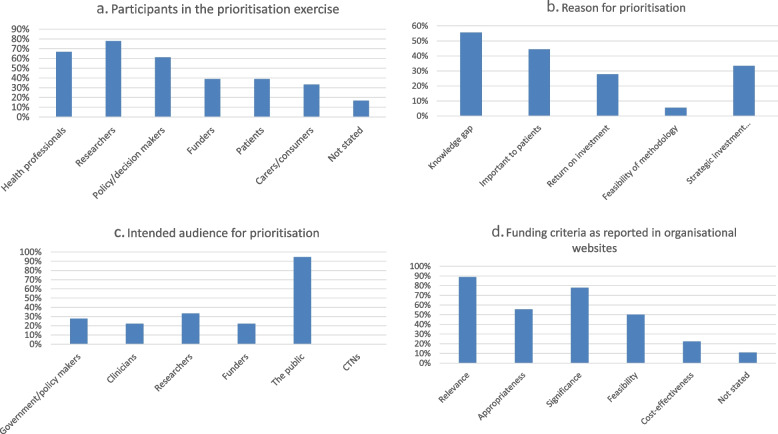
Research prioritisation participants, reasons, intended audience and funding criteria from websites of research funders and clinical trial networks. **a** Participants in the prioritisation exercise. **b** Reason for prioritisation. **c** Intended audience for prioritisation. **d** Funding criteria as reported in organisational websites

Thirty-nine websites of research funding organisations and CTNs were reviewed (Appendix), and 18 were found to contain research prioritisation information: one from Australia (6%), two from New Zealand (11%), one from Ireland (6%), eight from Canada (44%) and six from the USA (33%; see Table 3). A table of the clinical disciplines involved is depicted listed in Table 3. The stakeholders most frequently involved in priority setting were researchers (*n* = 14 websites; 78%) followed by health professionals (*n* = 12 websites; 67%) and policy/decision makers (*n* = 11 websites; 61%; see Fig. 4a). Funders and patients were mentioned in seven processes each (39%) and carers/consumers were mentioned six times (33%). Participants or stakeholders were not stated in three occasions (17%; see Fig. 4a).

**Table 3:** Websites searched

	***Citation***	***Country***	***Author group (CTN, funder)***	***Clinical discipline***
1	Framework for Identification and Prioritisation of Targeted Calls for Research	Australia	NHMRC	Health and Medical Research
2	National Science Challenge description	New Zealand	MBIE	All
3	New Zealand Health Research Prioritisation Framework	New Zealand	HRC	All
4	Cross-department priorities	Ireland	Research Prioritisation Project Steering Group	All
5	SPOR Patient engagement framework	Canada	CIHR	All
6	Institute for Musculoskeletal Health and Arthritis	Canada	IMHA	Musculoskeletal Health and Arthritis
7	IMHA strategic plan 2014-2018	Canada	IMHA	Musculoskeletal Health and Arthritis
8	IMHA Priority setting - 2018-2020 National Listening tour	Canada	IMHA	Musculoskeletal Health and Arthritis
9	IMHA fibromyalgia case study	Canada	IMHA	Fibromyalgia
10	Institute for Circulatory and Respiratory Health - ICRH strategic plan 2020	Canada	ICRH	Circulatory and Respiratory Health
11	Institute for Population and Public Health – IPPH listening tour 2016	Canada	IPPH	All
12	Institute for Population and Public Health – strategic plan 2009-2014	Canada	IPPH	All
13	PCORI Methodology report	US	PCORI	All
14	PCORI-Generation and Prioritization of Topics for Funding Announcements	US	PCORI	All
15	NIH Strategic plan 2016-2020	US	NIH	All
16	NIMH Strategic plan 2020	US	NIMH	Mental Health
17	NCBI: Priorities in Health 2006	US	The World Bank, WHO, Fogarty International Center NIH	All
18	NHLBI priority-setting process	US	NHLBI	All

*CIHR* Canadian Institutes of Health Research, *HRC* Health Research Council of New Zealand, *CTN* Clinical trials network, *ICRH* Institute for Circulatory and Respiratory Health, *IMHA* Institute for Musculoskeletal Health and Arthritis, *IPPH* Institute for Population and Public Health, *MBIE* Ministry of Business, Innovation and Employment, *NCBI* National Center for Biotechnology Information, *NHLBI* National Heart, Lung, and Blood Institute, *NHMRC* National Health and Medical Research Council, *NIH* National Institutes of Health, *NIHR* National Institute for Health Research, *NIMH* National Institute of Mental Health, *PCORI* Patient-Centered Outcomes Research Institute, *SPOR* Strategy for Patient-Oriented Research, *WHO* World Health Organization

A “knowledge gap” was the reason for developing a prioritisation guideline among 10 websites (56%), followed by “wanting to know what was important to patients” (*n* = 8 websites; 44%). Six organisations mentioned the reason for the priority-setting exercise was to support their vision and mission or to invest strategically and in a balanced way (33%) and five organisations wanted to find the best return on investment (32%; see Fig. 4b). Feasibility of the methodologies used was mentioned once (6%).

The intended audience was in all but one case the general public (*n* = 17 websites; 94%), followed by the researchers (*n* = 6 websites; 33%), the government and policymakers (*n* = 5 websites; 28%) and clinicians or funders (*n* = 4 websites each; 22%; see Fig. 4c).

As for the prioritisation tools used, three organisations used workshop/consensus meeting (17%), one used the JLA tool, one used payback (VOI) and one used MCDA (6%). Five organisations used other tools (surveys, working groups; 28%) and seven did not describe the tool used (39%). In general, few details were provided in organisational websites to further describe the prioritisation approaches undertaken.

The prioritisation criteria included relevance on 16 occasions (89%), appropriateness on 10 occasions (56%), significance on 14 occasions (78%), feasibility on 9 occasions (50%) and cost-effectiveness on 4 occasions (22%). Two websites did not state any prioritisation criteria (11%; see Fig. 4d).

## Discussion

This extensive scoping review summarises findings from international agencies about current methods and approaches to prioritisation of clinical trials undertaken by CTNs and research funders. The main reasons for prioritisation were to address a knowledge gap in clinical decision making, and to define patient-important topics. More than two thirds used an interpretive approach (e.g. James Lind Alliance); a small proportion used a quantitative approach (e.g. prospective payback); and one fifth used a blended approach combining qualitative and quantitative methods (e.g. CHNRI). The most common criteria for prioritisation were significance, relevance and cost-effectiveness.

The rationale for prioritisation of trials on the basis of generating new knowledge to improve clinical decision-making is not surprising, as efficacy and effectiveness trials are designed to answer important questions in patient management [27, 50, 88]. What was less clear, however, was how these trials all with “good” questions were then ranked in order of priority. Consensus-based methods that use an interpretative approach are appealing because of their broad stakeholder engagement; however, the trade-offs between criteria, such as significance versus feasibility, and the subsequent processes for overall ranking of trials are not transparent [89, 90]. This is where blended approaches that include a quantitative component that facilitates objective scoring of trial proposals can assist.

The infrequent use of pure quantitative approaches for prioritisation of trials including burden of disease or value for money is likely due to few standardised methods to view competing claims side by side, or knowing how to weight such criteria. It may also be related to low technical knowledge or expertise within the trials community to generate this information. For example, many clinical trial funders suggest the relevance of the problem to be stated, which is typically reported as burden of disease, incidence or prevalence. When different metrics are used across trial proposals they become difficult to compare, which may lead to grant reviewers considering whether the criterion is satisfied (i.e. is there a substantial burden, [yes/no]), rather than comparing those burdens. Sometimes, the burden is presented as disability-adjusted life years (DALYs), and sometimes, the disease burden is monetized to provide an overview of health system or societal costs. While this provides a common metric on which trial applications can be compared, these estimates are limited to quantifying the current situation; they do not provide insight into the value of the proposed trial in reducing that burden (i.e. the significance), otherwise known as the impact or net benefit.

Value of information (VOI) analysis has emerged as a new framework for quantifying the net benefit of proposed randomised trials. VOI uses a cost-effectiveness modelling approach and takes into account the cost of running the trial and the value of the new trial information to reduce uncertainty with the current clinical decision. The benefit of the health outcomes for the better decision (e.g. using drug A over drug B) is then multiplied across the population at risk using assumptions about post-trial implementation. A VOI analysis can be undertaken for most randomised trials enabling studies in a given portfolio to be ranked from most to least value. This requires capacity building in the health economics and statistics workforce. Efficient methods to calculate VOI are currently underway [91, 92].

An encouraging sign from this review was the emphasis placed on patient-important topics through consumer-generated questions and topic ranking, from both published literature and organisational websites. This ensures that not only are questions important and of interest to clinicians or trialists, but that they also address issues, problems or concerns that are bothering those with the disease and/or undergoing specific treatments. This is especially important for government and non-profit charity funders where the funding for research originates from the general public (i.e. tax-payers), or donors.

The strengths of this review include the dual searching of published and unpublished literature, including organisational websites of international clinical trial networks and trial funders. This approach was likely to identify prioritisation processes that were operational, yet had not been formally described in the peer-reviewed literature. It is a strength that we were able to locate and extract research prioritisation approaches and methods as well as the prioritisation criteria used, as this provides sufficient detail for clinical trials networks and funders to replicate. Our scoping review was limited to studies and websites published in English and therefore may omit relevant studies published in other languages. It was not a systematic review and therefore may not have identified all studies of research prioritisation in the published literature. In addition, we could only tabulate methods where they were clearly described.

Further research consulting consumers, researchers and policy-makers is now needed to develop specific criteria weights for clinical trials networks and coordinating centre members of the Australian Clinical Trials Alliance (ACTA), of international CTNs and funders of clinical trials. Development of tools to aid clinicians and researchers in using quantitative approaches is also needed. Following implementation of a formalised prioritisation process, clinical trials networks and funders will need to then evaluate the process and assess whether the “best” trials are subsequently funded and deliver on their expected benefits [93].

## Conclusion

Research prioritisation approaches for groups conducting and funding clinical trials are predominantly interpretative. Given the strengths of a blended approach to prioritisation, there is an opportunity to improve the transparency of process through the inclusion of quantitative techniques.

## Data Availability

Data generated and analysed during this study are included in this published article and its supplementary information files.

## Appendix

### Key Australian and international clinical trials networks (CTNs) /clinical disciplines/ clinical specialties and Funders’ websites

#### Clinical trials network websites

**Table:**

▪ Australia & New Zealand Musculoskeletal (ANZMUSC) Clinical Trials Network ▪ Australasian College for Emergency Medicine (ACEM) Clinical Trials Group ▪ Australasian Gastro-Intestinal Trials Group (AGITG) ▪ Australasian Lung Cancer Trials Group (ALTG) ▪ Australasian Radiopharmaceutical Trials Network (ARTNET) ▪ Australasian Sarcoma Study Group (ASSG) ▪ Australasian Society for Infectious Diseases (ASID) Clinical Research Network ▪ Australasian Stroke Trials Network (ASTN) ▪ Australia & New Zealand Breast Cancer Trials Group (ANZBCTG) ▪ Australia & New Zealand Melanoma Trials Group (ANZMTG) ▪ Australia New Zealand Gynaecological Oncology Group (ANZGOG) ▪ Australian & New Zealand Children’s Haematology/Oncology Group (ANZCHOG) ▪ Australian & New Zealand College of Anaesthetists (ANZCA) Clinical Trials Network ▪ Australian & New Zealand Intensive Care Society (ANZICS) Clinical Trials Group ▪ Australian & New Zealand Urogenital & Prostate (ANZUP) Cancer Trials Group ▪ Australian Epilepsy Clinical Trials Network (AECTN) ▪ Australian Paediatric Research Network (APRN) ▪ Australian Primary Care Research Network (APCReN) ▪ Cooperative Trials Group for Neuro-Oncology (COGNO) ▪ Multiple Sclerosis Research Australia Clinical Trials Network (MS Australia) ▪ NSW Better Treatments 4 Kids (BT4K) ▪ Paediatric Research in Emergency Departments International Collaborative (PREDICT) ▪ Paediatric Trials Network Australia (PTNA) ▪ Palliative Care Clinical Studies Collaborative (PaCCSC) ▪ Primary Care Collaborative Cancer Clinical Trial Group (PC4) ▪ Psycho-Oncology Co-operative Research Group (PoCoG) ▪ The Australasian Consortium of Centres for Clinical Cognitive Research (AC4R) ▪ The Australasian Kidney Trials Network (AKTN) ▪ The Australasian Sleep Trials Network (ASTN) ▪ The Spinal Cord Injury Network (SSCIS) ▪ The Australian Type 1 Diabetes Clinical Research Network (T1DCRN) ▪ The Interdisciplinary Maternal Perinatal Australasian Collaborative Trials Network (IMPACT) ▪ Therapeutic and Vaccine Research Program, Kirby Institute ▪ Trans-Tasman Radiation Oncology Group (TROG)	

#### Funders’ websites

National Health and Medical Research Council (NHMRC)—Australia

Medical Research Future Fund (MRFF)—Australia

Medical Research Council (MRC)—United Kingdom

National Institute for Health Research (NIHR)—United Kingdom

Health Research Board (HRB)—Ireland

Canadian Institutes for Health Research (CIHR)—Canada

National Science Challenges (NSCs)—New Zealand

Patient-Centered Outcomes Research Institute (PCORI)—United States James Lind Alliance Evidence Gap Maps

Health Research Council of New Zealand—New Zealand

National Institutes of Health (NIH)—United States

Department of Business, Enterprise and Innovation—Ireland

## Abbreviations

ACTA: Australian Clinical Trials Alliance
AHRQ: Agency for Healthcare Research and Quality
AKTN: Australasian Kidney Trials Network
ANZCA: Australian and New Zealand College for Anaesthetists
ANZIC-RC: Australian and New Zealand Intensive Care Research Centre
ANZICS: Australian and New Zealand Intensive Care Society
CHNRI: Child Health Nutrition Research Initiative
CTN: Clinical Trial Network
DALY: Disability-adjusted life years
EVI: Expected Value of Information
JLA: James Lind Alliance
MCDA: Multi-criteria Decision Analysis
NHMRC: National Health and Medical Research Council
PPoR: Prospective Payback of Research
ROI: Return on investment
VOI: Value of information
WHOLIS: World Health Organization Library Database
